# Using light exposure to improve sleep and circadian entrainment for people with dementia

**DOI:** 10.1002/alz70858_104438

**Published:** 2025-12-26

**Authors:** Erik Page

**Affiliations:** ^1^ Blue Iris Labs, Fairfax, CA, USA

## Abstract

**Background:**

People with AD/ADRD are often trapped in a vicious cycle: their disease interferes with sleep, and poor sleep exacerbates their cognitive decline. Light exposure, the primary regulator of circadian rhythms, has a significant impact on AD/ADRD patients: people who get “good” light exposure (bright days and dark evenings/nights) experience better sleep and reduced cognitive decline (among other positive health outcomes) compared to those who get “bad” light (dim days and bright nights). However, due to technological limitations–accurate spectrometers have historically been too large to use outside of controlled experimental settings–our understanding of the light‐health relationship in regular living has been limited, and light detection systems for personal, non‐pharmacological interventions have been out of the question.

**Method:**

Our study deployed the Blue Iris Speck wearable spectrometer with healthy adults for 30+ days to study relationships between light and sleep. Participants (*N* = 28; ages 25 to 81), agreed to wear the Speck and track their sleep for 30‐50 days. This pilot study aimed to refine protocols for future long‐term trials of a light exposure wearable and app‐based “light coach” for AD/ADRD populations, and to develop approaches for data cleaning and analysis, with particular interest in detecting whether the participants were wearing the Speck consistently.

**Results:**

Technically, the Speck wearable performed well, with extensive light‐exposure data for at least 30 days for 27 of 28 participants. User compliance was also good: all but one of the participants remained active for 30 days, and most continued for 20 or more extra days. Analyses to detect user compliance showed patterns of wear/non‐wear similar to expectations, suggesting the method's potential. Analyses to detect the light exposure‐sleep relationship showed that, despite inter‐individual variation, light early in the day was positively correlated with good sleep and light at night was negatively correlated with good sleep.

**Conclusion:**

A wearable light detection system was effectively deployed for an extended period, with good data reporting throughout. User compliance with wearing the spectrometer was good enough that there is reason to hope AD/ADRD patients will also find it easy to use a wearable for both research and personal health.

